# Chronological age-related metabolome responses in the dinoflagellate *Karenia mikimotoi*, can predict future bloom demise

**DOI:** 10.1038/s42003-023-04646-z

**Published:** 2023-03-15

**Authors:** Takeshi Hano, Yuji Tomaru

**Affiliations:** Environment Conservation Division, Fisheries Technology Institute, National Research and Development Agency, Japan Fisheries Research and Education Agency, 2-17-5 Maruishi, Hatsukaichi, Hiroshima, 739-0452 Japan

**Keywords:** Plant physiology, Plant ecology

## Abstract

*Karenia mikimotoi* is a common harmful algal bloom (HAB)-forming dinoflagellate and has caused severe financial loss in aquaculture. There are limited metabolomic studies on dinoflagellate biology. Here, we examined alterations in metabolic profiles over the growth curve of *K. mikimotoi* under nitrogen or phosphorus deficiency and further explored a key criterion for the diagnosis of late stationary phase to identify when the dinoflagellate cells will enter bloom demise. The results demonstrate the differential expression of metabolites for coping with chronological aging or nutrient deprivation. Furthermore, an increase in the glucose to glycine ratio in the late stationary phase was indicative of dinoflagellate cells entering bloom demise; this was also detected in the cultured diatom, *Chaetoceros tenuissimus*, indicating that this may be the general criterion for phytoplankton species. Our findings provide insights regarding chronological aging and the criterion for the prediction of phytoplankton bloom demise.

## Abstract (web summary)

A broad survey of the metabolome over the growth curve of K. mikimotoi identifies glucose to glycine ratio as indicative of dinoflagellate cells entering the late stationary phase, predicting bloom demise.

## Introduction

Phytoplanktons are the dominant primary producers of marine ecosystems and their blooms vary temporally and spatially^1,2^. In the past few decades, the increase in the frequency and distribution of harmful algal blooms (HABs) in marine ecosystems, caused by environmental fluctuations such as variations in nutrient levels^3,4^, has led to closures of commercial fisheries and aquaculture operations, resulting in considerable economic losses^5^. Nitrogen and phosphorus are essential macronutrients that are involved in fundamental metabolic processes in phytoplanktons^6,7^. Consequently, N and P concentrations influence the population dynamics and physiology of harmful algae at the species level^6,8,9^.

*Karenia mikimotoi* is one of the most common HAB-forming dinoflagellate species that has severely damaged the aquaculture industry by causing mortality of fish and shell fish in the coastal areas of Europe and Asia^10^. In the coastal areas of western Japan, red tides of *K. mikimotoi* led to huge financial loss in aquaculture, amounting to ~15 million USD in 2012^11^. N or P deprivation impairs the growth of the dinoflagellate; however, they have adopted various strategies to maintain their population, and their complex modes of alimentation support widespread distribution and survival in unfavorable environments^10,12^. These strategies include adaptation to eurythermal and euryhaline environments^13^, temporal cyst formation under N deficiency^12^, and the ability to utilize various forms of N or P^12,14–16^. Furthermore, with the advent of genomic-, transcriptomic-, and proteomic-based approaches, our understanding of dinoflagellate biology has progressed in the past decade^6,7,17,18^.

Of particular interest, but largely unknown in dinoflagellate biology, is the cellular process of chronological aging over the growth curve, defined as the processes a cell undergoes to survive in exponential, stationary, and decline phases. Furthermore, understanding the processes regulating chronological aging will enhance our knowledge regarding the ability of *K. mikimotoi* blooms to persist in the coastal environment. However, information about the later aging processes, such as from late stationary phase to decline phase, is scarce in the available literature. The future bloom demise forecast can be possibly achieved by investigating the key criteria associated with late stationary phase, which is highly required in the field of aquaculture.

Metabolomics has been recognized as an important “omics” science for characterizing endogenous low-molecular-weight metabolites present in any biological system^19^. This approach is a powerful tool for investigating the metabolism of organisms, including algae, and for filling the phenotype-genotype gap because it presents a closer picture of cellular activity than other omics methods^20,21^. Although the application of metabolomics to dinoflagellates is relatively new, it has already contributed considerably to dinoflagellate biology^5,20^. However, so far, information regarding metabolomics research for understanding dinoflagellate biology under nutrient deprivation is scarce, and the metabolic mechanisms involved in the aging process that may precede the culture demise remain unexplored.

Hence, in this study, we performed a broad survey of the metabolome over the growth curve of *K. mikimotoi* to obtain insights into the adaptation to nutrient (nitrogen or phosphorus) limitation and chronological aging, defined here as a process that underlies entry into culture demise. The results suggested the presence of differential expression of metabolites for key processes involved in energy production, photosynthesis, and carbon metabolism and that metabolic restructuring is required for coping with conditions that occur during chronological aging or nutrient deprivation. Among the metabolites identified in *K. mikimotoi*, the ratio of glucose to glycine was remarkably increased in cells in the late stationary phase, which is indicative of dinoflagellate cells entering into bloom demise. The present findings provide insights regarding chronological aging, which were obtained by monitoring key indexes for traits such as prediction of bloom demise.

## Results

### Growth

**Fig. 1:**
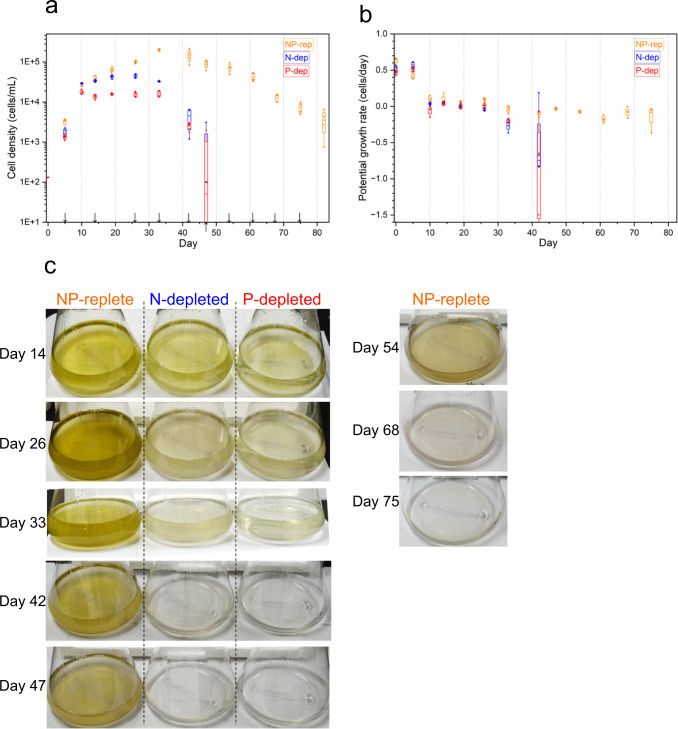
Biological and morphological changes in the *K. mikimotoi*. Time - course changes of cell density (cells per milliliter) (**a**), potential growth rate (PGR) (**b**), and representative appearance of each culture medium (**c**). In (**a**–**c**), The dinoflagellate cells were cultured for 47 or 82 days in the SWM-3 medium with different concentrations of nitrogen (NaNO_3_) and phosphate (NaH_2_PO_4_) and initial N and P concentrations were 2 and 0.1 mM for NP-replete (NP-rep), 0.2 and 0.1 mM for N-depleted (N-dep), and 2 and 0.0033 mM for P-depleted (P-dep) treatment groups. For box plots (**a**, **b**), horizontal lines indicated median, top and bottom ends of vertical bars indicated maximum and minimum data points within 1.5 inter-quartile range (*n* = 4). In (**a**), arrows indicate the sampling point for metabolomics. Rep replete, dep depleted.

Changes in *K. mikimotoi* cell density were present throughout the complete growth curve in all treatment groups, including the exponential, stationary, and decline phases (Fig. 1a). The mean maximum cell density was 2.0 × 10^5^ cells per milliliter, 4.4 × 10^4^ cells per milliliter, and 1.7 × 10^4^ cells per milliliter for NP-replete (NP-rep), N-depleted (N-dep), and P-depleted (P-dep) treatment groups, respectively (Fig. 1a). Potential growth rate (PGR) exceeded 0.4 for the first 10 days, as cells in all treatment groups proliferated rapidly (Fig. 1b). Thereafter, it was maintained between −0.1 and 0.1, following which it dropped below −0.1 in both N-dep and P-dep groups by 47 days (Fig. 1b).

The cell size of *K. mikimotoi* during the experimental period is shown in Supplementary Fig. 1. The dinoflagellate cells in NP-rep groups showed a constant cell size until day 61; however, algal cell size in both N- and P-dep treatments were enlarged and thereafter gradually decreased as they aged. As a result, a significant increase in cell size relative to those of NP-rep treatments were observed over 14–33 and 9–42 days, for N- and P-dep treatments, respectively.

In all treatment groups, the chlorophyll red fluorescence intensity was the highest (~12,000) at around 10 days (Supplementary Fig. 2). Thereafter, in the NP-rep treatment, it decreased gradually to levels that were ~80% of the apical value. The fluorescence intensity decreased dramatically after N-dep and P-dep treatments to ~50% of the maximum values. Furthermore, when comparison was made based on the same sampling date, significant decrease of N-dep and P-dep treatments relative to NP-rep group was observed over 19–42 days except for P-dep treatment on day 33.

As shown in Fig. 1c, a remarkable dark and ochreous culture medium was observed in the NP-rep group over 26–42 days, and the medium gradually turned colorless as the number of cultured cells started to decrease. The color of the culture medium in the nutrient starvation groups was fainter than that in the NP-rep group, and it was colorless on days 42 and 47.

### Metabolic alterations in dinoflagellate cells over the growth curve

**Fig. 2:**
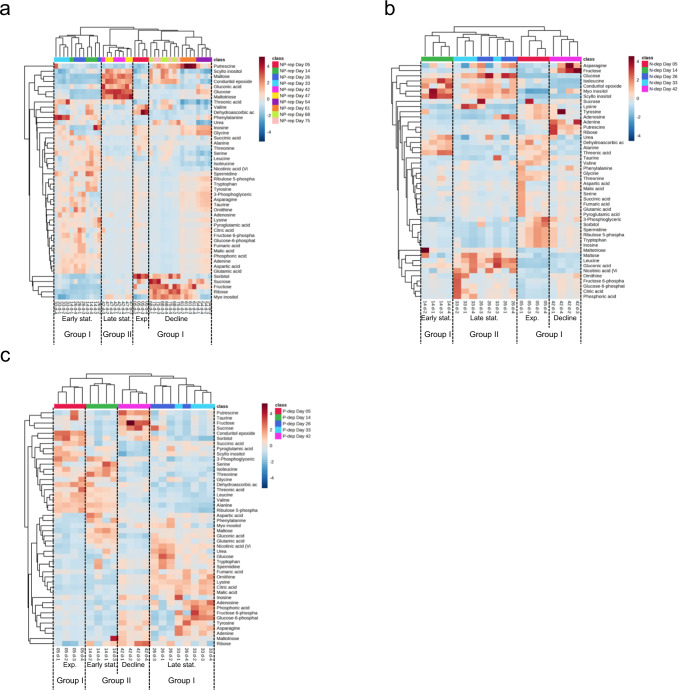
Hierarchical heatmap showing the metabolic changes in the *K. mikimotoi*. **a** NP-replete; **b** N-depleted; and **c** P-depleted experimental groups. The dinoflagellate cells were cultured for 47 or 82 days in the SWM-3 medium with different concentrations of nitrogen (NaNO_3_) and phosphate (NaH_2_PO_4_) and initial N and P concentrations were 2 and 0.1 mM for NP-replete (NP-rep), 0.2 and 0.1 mM for N-depleted (N-dep), and 2 and 0.0033 mM for P-depleted (P-dep) treatment groups. Log_2_ fold changes are shown with a color scale with dark red and dark blue indicate significant increase and decrease. Individual samples are separated using hierarchical clustering, with the dendrogram scaled to represent the distance between each branch. Group I, exponential, early stationary and decline phase; Group II, late stationary phase. Exp. exponential, stat. stationary.

We successfully identified 46 water soluble metabolites from cultured *K. mikimotoi*. Figure 2 shows the metabolic features over the growth curve of *K. mikimotoi*. Hierarchical clustering analysis clearly showed that metabolic fluctuation was dependent on growth phase, as indicated by four clearly delimited clusters corresponding to exponential (5 days for all treatments), early stationary (14–33 days for NP-rep and 14 days for N- or P-dep groups), late stationary (42–47 days for NP-rep and 26–33 days for N- or P-dep groups), and decline phases (54–75 days for NP-rep and 42 days for N- or P-dep groups).

**Fig. 3:**
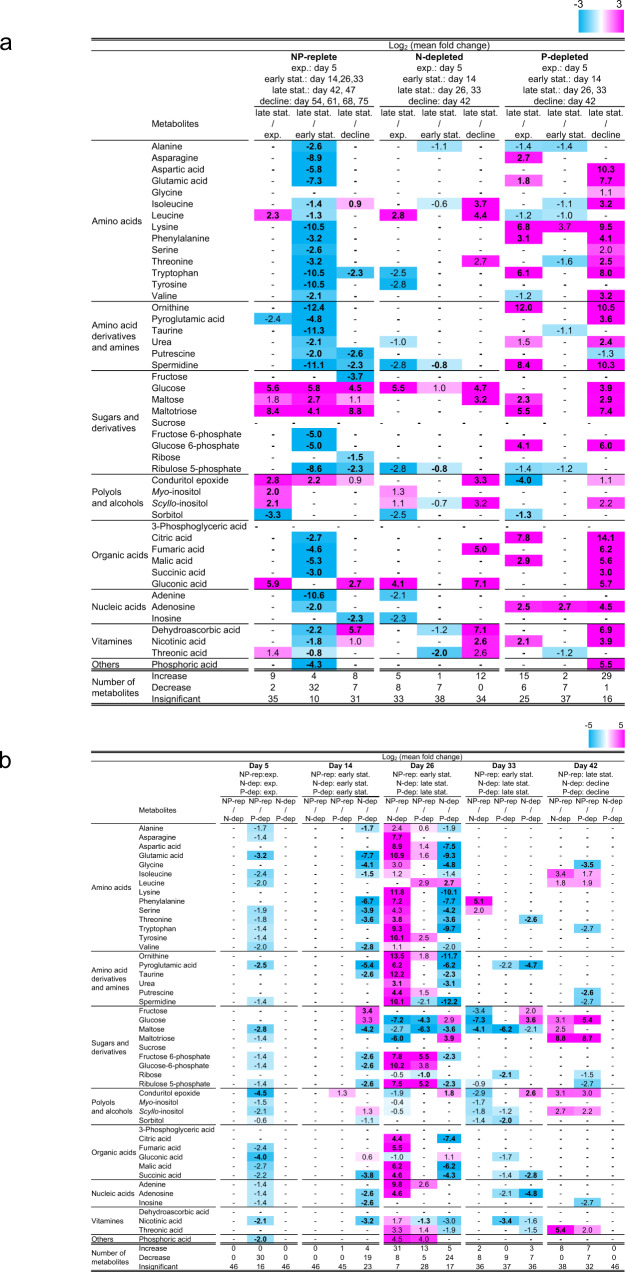
Heatmap of nutrient-, growth phase-, and age-related metabolic changes in the *K. mikimotoi*. **a** Log_2_-transformed mean fold changes in metabolites over the experimental period in various nutrient media. **b** Log_2_-transformed mean fold changes in metabolites in various nutrient media at each sampling date. The dinoflagellate cells were cultured for 47 or 82 days in the SWM-3 medium with different concentrations of nitrogen (NaNO_3_) and phosphate (NaH_2_PO_4_) and initial N and P concentrations were 2 and 0.1 mM for NP-replete (NP-rep), 0.2 and 0.1 mM for N-depleted (N-dep), and 2 and 0.0033 mM for P-depleted (P-dep) treatment groups. In **a** and **b**, plain and bold numbers indicate significant fold changes based on *t*-tests for parametric data and Welch’s *t*-tests for non-parametric data, respectively, after FDR adjustment at a significance threshold of 0.05 (*q* < 0.05). Fold changes are color-coded; pink and sky blue indicate significant increase and decrease and “-“denote insignificant changes. Exp. exponential, stat. stationary, rep replete, dep depleted.

We then determined the metabolic features of the dinoflagellate cells that entered culture decline. Toward this, the exponential, early stationary, and decline phases were compared with the late stationary phase. Figure 3a shows the log_2_ fold changes in metabolites between late stationary phase and the other three phases. Notably, alterations in metabolite levels varied substantially among the treatment groups. In the NP-rep treatment group, levels of amino acids and their derivatives decreased remarkably in the late stationary phase than in the early stationary phase (Fig. 3a). In contrast, the levels of *myo*- and *scyllo*-inositol or their derivative (conduritol epoxide) increased significantly in the late stationary phase than in the other phases. In addition, the levels of sugars (glucose, maltose, and maltotriose) in the late stationary phase were consistently higher than those in any other phases. In the N- and P-dep treatment groups, glucose levels in the late stationary phase also remained higher than those in the other phases, and overall, the levels of metabolites between early and late stationary phases differed negligibly (Fig. 3a). In the N-dep treatment group, the number of differential metabolites between 14 days (early stationary phase) and 26–33 days (late stationary phase) was less, whereas the levels of 12 metabolites in the N-dep treatment group at 26–33 days were consistently higher than those at 42 days (decline phase). In the P-dep treatment group, the levels of the majority of metabolites, including amino acids, sugars, and organic acids, increased at 26–33 days, compared to those observed at 5 days and 42 days, in contrast to minimal changes at 14 days.

As shown in Supplementary Fig. 3, N or P starvation substantially influenced the metabolic content in dinoflagellate cells even in the same growth phase. In the exponential phase (day 5), which corresponds to the phase when phytoplankton cells in all treatment groups achieved similar growth rate (Fig. 1b), the levels of 29 metabolites in the NP-rep group were significantly lower than those in the P-dep treatment group and their amounts followed the order P-dep ≥ N-dep ≥ NP-rep. In the early stationary phase, most of the metabolite levels in the N-dep treatment group were remarkably lower than those in the other treatment groups. However, the metabolic profile differed slightly in the late stationary phase between N-dep and NP-rep groups, and P-dep had higher metabolic content than NP-rep and N-dep. One exception in early and late stationary phases was that glucose level was higher in the N-dep treatment group than in any other treatment groups. In the decline phase, fewer number of metabolites differed among the treatments. Similarly, comparison of metabolites on the same sampling date (Fig. 3b) showed the same trends as those shown in Supplementary Fig. 3; the metabolic contents in *K. mikimotoi* cells under N or P deficiency were distinct in stationary phases (14 days and 26 days), but similar within the exponential and decline phases (5 days and 42 days).

### Exploration of candidate metabolites associated with the late stationary phase

**Fig. 4:**
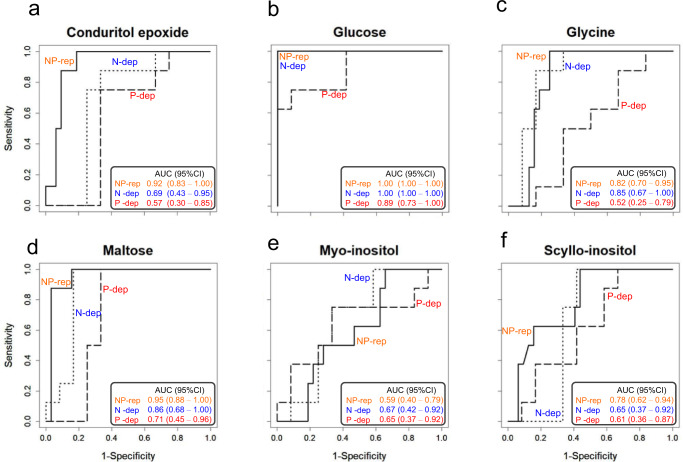
Receiver operating characteristic (ROC) curve of specified metabolites in the *K. mikimotoi*. Of the 46 metabolites tested, six candidate metabolites (**a**–**f**) were selected whose mean signal-to-noise ratio (S/N ratio) being higher than 20 per 1 × 10^6^ cells per milliliter for any treatment group and sampling date. The dinoflagellate cells were cultured for 47 or 82 days in the SWM-3 medium with different concentrations of nitrogen (NaNO_3_) and phosphate (NaH_2_PO_4_) and initial N and P concentrations were 2 and 0.1 mM for NP-replete (NP-rep), 0.2 and 0.1 mM for N-depleted (N-dep), and 2 and 0.0033 mM for P-depleted (P-dep) treatment groups. In (**a**–**f**), straight line; NP-replete, dotted line; N-depleted, and dashed line; P-depleted, and column in each box shows area under the curve (AUC) with 95% credible interval (CI). Group I, exponential, early stationary, and decline phase; Group II, late stationary phase. Rep replete, dep depleted.

Using the 46 metabolites identified, we first selected candidate metabolites that were robustly detected in dinoflagellate cells with the mean signal-to-noise ratio (S/N ratio) being higher than 20 per 1 × 10^6^ cells per milliliter for any treatment group and sampling date. As a result, six metabolites were selected, namely conduritol epoxide, glucose, glycine, maltose, *myo*-inositol, and *scyllo*-inositol (Supplementary Fig. 4). Receiver operating characteristic (ROC) analysis was subsequently performed with the six metabolites between the two specified groups, Group I (exponential, early stationary, and decline phases) and Group II (late stationary phase). Among them, glucose had higher area under the curve (AUC; with 95% credible interval (CI))values than the other five metabolites, and it was the only metabolite exceeding the threshold of 0.7 in all treatment groups (Fig. 4 and Supplementary Table 1). This indicated that glucose level increased exclusively in Group II (Supplementary Fig. 4) and could be a potential biomarker associated with the late stationary phase.

To improve the accuracy of prediction over that obtained using a single compound (glucose), we individually calculated the ratio of glucose level relative to that of five other metabolites (Supplementary Table 2). ROC analysis was again performed using the specified five ratios between Groups I and II. AUC (with 95% CI) yielded satisfactory values for the ratios of glucose to conduritol epoxide, glycine, *myo*-inositol, or *scyllo*-inositol (Supplementary Table 2), and these were significantly higher in Group II than in Group I (Table 1). Furthermore, the fold change of the glucose to glycine ratio was higher than that of the other specified ratios in all treatment groups (Table 1), which improved ROC outcomes (Supplementary Tables 1 and 2). Therefore, we concluded that the glucose to glycine ratio is a highly credible candidate marker that facilitates prediction of the late stationary growth phase of phytoplanktonic cells.

**Table 1:** Mean fold changes in the ratio of glucose to the other five candidate metabolites.

	NP-replete^a^			N-depleted^a^			P-depleted^a^		
	Group I	Group II	Mean fold change (Group II/I)	Group I	Group II	Mean fold change (Group II/I)	Group I	Group II	Mean fold change (Group II/I)
Glucose/Conduritol epoxide	3.8 ± 4.0	28 ± 3.8**	7.3	23 ± 20	86 ± 44**	3.7	12 ± 15	39 ± 14**	3.4
Glucose/Glycine	4.7 ± 9.0	240 ± 60**	51	88 ± 120	760 ± 390**	8.7	1.0 ± 0.7	3.8 ± 1.5**	3.7
Glucose/Maltose	5.7 ± 4.7	44 ± 8.2**	7.6	40 ± 44	91 ± 50*	2.3	1.0 ± 0.9	1.2 ± 0.42**	1.2
Glucose/*Myo*-inositol	1.6 ± 1.6	35 ± 9.1**	22	15 ± 17	79 ± 14**	5.4	3.0 ± 2.2	8.7 ± 1.8**	2.9
Glucose/*Scyllo*-inositol	0.074 ± 0.078	1.1 ± 0.14**	15	0.61 ± 0.39	2.6 ± 0.46**	4.3	0.14 ± 0.070	0.38 ± 0.14**	2.8

^a^:* and ** denote significant difference between Group I and II at *p* < 0.05 and *p* < 0.01, respectively (two-sample *t*-test). Group I, exponential, early stationary and decline phase; Group II, late stationary phase.

**Fig. 5:**
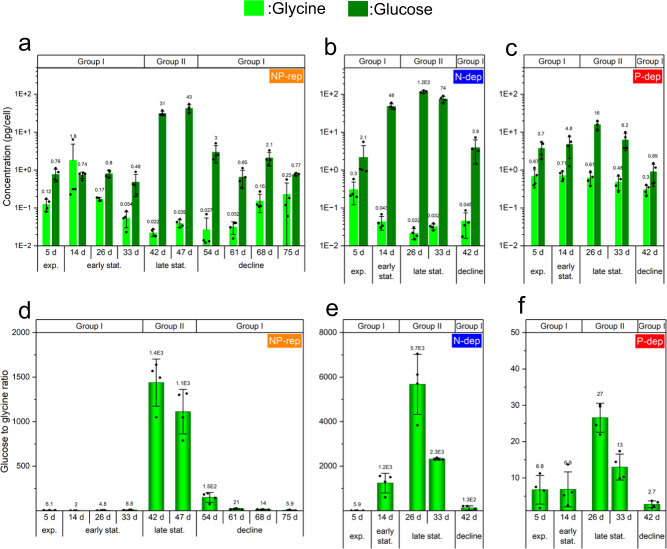
Chronological changes of candidate metabolites and criterion in the *K. mikimotoi*. In (**a**–**c**), concentration (pg per cell) of two candidate metabolites (glucose and glycine) and criterion (ratio of glucose to glycine) (**d**–**f**). The dinoflagellate cells were cultured for 47 or 82 days in the SWM-3 medium with different concentrations of nitrogen (NaNO_3_) and phosphate (NaH_2_PO_4_) and initial N and P concentrations were 2 and 0.1 mM for NP-replete (NP-rep), 0.2 and 0.1 mM for N-depleted (N-dep), and 2 and 0.0033 mM for P-depleted (P-dep) treatment groups. Rep replete, dep depleted. Data represent mean ± standard deviation (SD; *n* = 4).

We then measured glucose and glycine concentrations in the dinoflagellate cells. The average glucose concentration ranges were 0.48–115 pg per cell, and glycine concentrations were relatively lower than those of glucose (0.022–1.8 pg per cell) (Fig. 5a–c). Consequently, the glucose to glycine ratios in Group II were 1113–1438, 2316−5676, and 13−27 for NP-rep, N-dep, and P-dep, respectively, and these were consistently higher than those in the Group I (Fig. 5d–f).

**Fig. 6:**
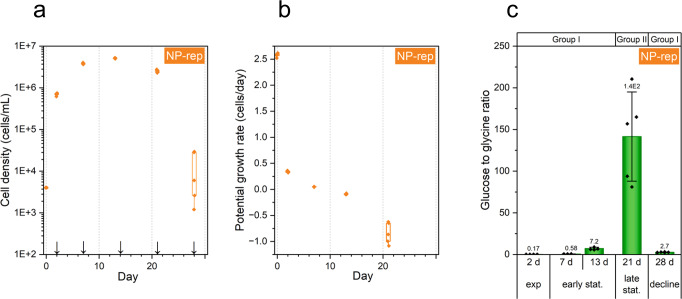
Chronological changes in the *C. tenuissimus*. Time - course changes of cell density (cells per milliliter) (**a**), potential growth rate (PGR) (**b**), and ratio of glucose to glycine (**c**). In (**a**–**c**), the diatom cells were cultured for 28 days in the SWM-3 medium and initial nitrogen (NaNO_3_) and phosphate (NaH_2_PO_4_) were 2 and 0.1 mM. For box plots (**a**, **b**), horizontal lines indicated median, top and bottom ends of vertical bars indicated maximum and minimum data points within 1.5 inter-quartile range (*n* = 5). In (**a**), arrows indicate the sampling point for metabolomics. In (**c**), Group I, exponential, early stationary and decline phase; Group II, late stationary phase. Exp, exponential; stat, stationary and note that glucose to glycine ratios were calculated from the relative abundance of each metabolite normalized to the cell density and internal standard. Data represent mean ± standard deviation (SD; *n* = 5). Rep replete.

Furthermore, we evaluated the metabolic profiles of the cultured marine diatom, *C. tenuissimus* NIES-3715, for 28 days under the NP-rep condition. The diatom cells showed chronological changes throughout the growth curve (Fig. 6a, b), and the glucose to glycine ratio was remarkably increased in the late stationary phase (day 21). This indicates that this ratio is a promising candidate for predicting the late stationary phase (i.e., onset of bloom demise) even in diatom species (Fig. 6c).

## Discussion

The present study clearly indicates changes in the microbial biomass of a dinoflagellate, *K. mikimotoi*, over the growth curve under NP-rep and N- or P-dep conditions. We also observed unique metabolic features in terms of cell aging, as well as nutrient deprivation in *K. mikimotoi* cells. Among those, we successfully identified a key metabolite signature for the late stationary phase, which allowed us to predict future bloom demise.

As shown in (Fig. 1a), consistently smaller cell density and relatively shorter bloom period in N- and P-dep groups than in the NP-rep group were associated with nitrogen or phosphorus exhaustion^6,7,12^. Global metabolomics analysis indicated that metabolic contents varied substantially according to the physiological state (Figs. 2 and 3) and that these classifications were consistent with those of cell density and PGR (Fig. 1b); PGR > 0.1 indicated the exponential phase; −0.1 < PGR ≤ 0.1 indicated the stationary phase, and PGR ≤ −0.1 indicated the decline phase. This study clearly demonstrates global changes in the metabolism of cultured dinoflagellates over the complete growth curve of *K. mikimotoi* and provides insights regarding the process mediating chronological aging in this organism.

The cells of *K. mikimotoi* in N-dep and P-dep groups showed larger size and lower red fluorescence intensity than those in the NP-rep group (Supplementary Figs. 1 and 2). The low cell density of *K. mikimotoi* in both nutrient-deficient groups may be compensated by its large size and high volume^22^. Furthermore, smaller size may confer NP-rep cells a competitive advantage over larger cells because of higher surface area-to-volume ratio and smaller thickness of the diffusion boundary layer^23^. Enlargement of cell size has been observed in dinoflagellate *K. mikimotoi*^16^, *Amphidinium carterae*^24^, and *Prorocentrum donghaiense* under P starvation^22^. In addition, *P. donghaiense* cells showed retardation of photosynthetic parameters (e.g., concentrations of chlorophyl a and carotenoid, Fv/Fm and rETR) under P and N deficiency^22^. Collectively, alteration in cell size or chloroplast conditions may be indicative of nutrient deficient stress in dinoflagellates.

In the NP-rep treatment group, alterations in the levels of amino acids, sugars, organic acids, and inositols were pronounced between late stationary phase and the other phases (Fig. 3a). Increase in amino acid levels in the exponential and early stationary phase relative to the late stationary phase may be attributed to the activation of cell metabolism and synthesis of large amounts of amino acids required for the production of new cells^25^, or protein degradation and release of energy via oxidation of amino acids^26^. In the present study, a significant increase in sugar levels was also found in the late stationary phase. As maltose and maltotriose are disaccharide and trisaccharide, respectively, these results indicated that total glucose pools accumulated more in the late stationary growth phases of cultured cells than in any other phases. Decrease in the levels of organic acids associated with the tricarboxylic acid (TCA) was also observed in the late stationary phase compared to those observed in the early stationary phase (Fig. 3a). Together with the lower accumulation of glucose in the early stationary phase, these alterations may account for acceleration of TCA cycle in response to high energy demand for growing dinoflagellate cells. Increase in *myo*-inositol level in the stationary phase of *K. mikimotoi* was consistent with that in *C. tenuissimus*^27^. *Myo*-inositol is the most widespread and extensively studied cyclitol in plants^28,29^, which is involved in signal transduction, membrane tethering, stress tolerance, and phosphorus storage^29^. In addition, plants break *myo*-inositol down to glucuronic acid to synthesize cell wall pectins. The significant increase in *myo*-inositol level at the late stationary phase may contribute to the developmental transition from cell division to cell maturation^27^. Although information on *scyllo*-inositol is scarce, this polyol has also been detected in diatoms^27^ and its amount in the dinoflagellate cells was relatively higher than that of *myo*-inositol (Supplementary Fig. 4e, f). This indicated that *scyllo*-inositol may play functionally important roles in the maintenance of dinoflagellate cells. Future studies will be necessary to elucidate the biological role of *scyllo*-inositol in phytoplanktons. Collectively, the accurate sensing of amino acid, sugar, and inositol levels is possibly an important factor for the efficient regulation of biosynthesis and catabolism in dinoflagellates as well as in diatoms^27^.

The present study clearly demonstrated that the dinoflagellate cells showed a distinct metabolic response to N or P starvation, indicating that they may use a different metabolic strategy to cope with nutrient limitation. In particular, the dinoflagellate cells had different amounts of metabolites under N or P deprivation and the extent of alterations was more conspicuous in the early and late stationary phase (Supplementary Fig. 3).

Nitrogen is an essential element for all phytoplankton species, as it is indispensable for the synthesis of proteins, nucleic acids, and chlorophylls, and N starvation leads to inhibition of population growth^6,12^. N limitation to 1/10th of the NP-rep condition led to significant fluctuation in metabolic contents, which was more evident after 26 days (Fig. 3b), whereas cell density-based observation indicated that onset of N starvation might occur between 14 days and 19 days when cell division was arrested (Fig. 1a, b). On 26 days, the levels of almost all amino acids (13 out of 14 amino acids) in the N-dep group were significantly lower than those in the NP-rep group (Fig. 3b), indicating that the dinoflagellate cells experienced severe N starvation and suppressed primary amino acid synthesis to save N source^6,17^. In previous studies with *K. mikimotoi*, transcripts associated with protein degradation, N-uptake and assimilation, and glycolysis were upregulated at around the onset of N starvation (0.08 mM relative to 1/10th of NP-rep condition)^6^. Furthermore, these studies demonstrated increase in the expression levels of TCA cycle-related genes, which may account for the decrease in the levels of TCA cycle-associated organic acids under N-dep condition at 26 days in this study (Fig. 3b). This indicates that N-deprived dinoflagellate cells may compensate for the loss of assimilatory power (generated by photochemical reactions) for carbon fixation because of downregulation of photosynthesis^6^.

Phosphate is also essential for microalgae growth, as it is required for most metabolic structures and functions^9^. Retardation of cell growth and division under phosphate starvation in this study was in agreement with the observations of a previous study on *K. mikimotoi*^7,9^. Under P deficiency, we found consistent increase in the level of differential metabolites relative to NP-rep treatment on day 5 (Fig. 3b), which was partially explained by upregulation of proteins associated with organic acid synthesis in P-limited *K. mikimotoi* cells^7^. In addition, significant depletion of sugar phosphates, such as fructose 6-phosphate and glucose 6-phosphate on day 26 (Fig. 3b), may indicate adaptive response to P deficiency^30,31^.

A diagnostic approach was used to identify the candidate metabolite by distinguishing the late stationary phase (Group II) from the other phases (Group I) (Fig. 4). Among the 46 metabolites identified in *K. mikimotoi* cells, we observed a remarkable increase in glucose level during the late stationary phase (Fig. 5a–c and Supplementary Fig. 4b). In dinoflagellates, photosynthesis is actively driven during the exponential phase; hence, the carbon fixation activity reduced as they aged^32^. Similarly, the phytoplankton cells utilize carbon resources (i.e., glucose) more actively during the exponential phase than in the stationary phase^32^. As the level of glucose could be calculated by subtracting the amount of glucose utilized (e.g., respiration) from that generated by carbon synthesis (photosynthesis and degradation of storage carbohydrate [starch, an α-1,4 glucan]), the highest levels of glucose in the late stationary phase indicated that carbon synthesis outcompeted carbon utilization. By contrast, glucose may be directly consumed and may not accumulate in growing, active cells in the exponential and early stationary phases^33^, consequently leading to relatively lower levels of glucose.

Interestingly, glucose concentrations in the cells in the late stationary phase were higher under N deficiency than any other treatments (Supplementary Fig. 3), although N deficiency was a stressor and interfered with carbon metabolism, as indicated by metabolic alterations and chlorophyll red fluorescence intensity. In the N-deprived algal cells, higher glucose levels were observed because of enhanced degradation of chrysolaminarinin in *Phaeodactylum tricornutm* cells^34^, and genes involved in glycolysis, fatty acid metabolism, and the TCA cycle were upregulated in *K. mikimotoi*^6^. Therefore, an increase in glucose levels may be a result of increased degradation of storage carbohydrates and a complementary response to N deficiency. The relatively lower levels of glucose in P-dep group than those in any other treatment group (Supplementary Fig. 3) may be attributed to the higher glucose demand for energy acquisition against P-limitation even in the late stationary phase.

Out of the 46 metabolites identified from dinoflagellate cells, only few are important as the hallmarks of the late stationary phase. The most important of these are glucose and glycine. Glycine level tended to be lower at the late stationary phase in NP-rep and N-dep treatments over the growth curve of the dinoflagellate, although the difference was not significant (Figs. 3a and 5a–c). Although the physiological role of glycine is poorly characterized in dinoflagellates, it is possibly synthesized via the photorespiratory glycolate pathway, similar to that in diatoms and higher plants^19,33^. Higher amount of glycine in the P-dep treatment group than in the NP-rep and N-dep treatment groups was also observed in the stationary phase (Supplementary Fig. 3), which had been found in diatoms under nutrient (Fe) limitation^19^.

Notably, we observed a remarkable increase in the ratio of glucose to glycine in the late stationary growth phase in the diatom, *C. tenuissimus*, as well as in the dinoflagellate, *K. mikimotoi* (Figs. 5d–f and 6c). We did not determine the specific ratio in *C. tenuissimus* under N or P deficiency; however, considering that the amounts of glucose and glycine were associated with fundamental physiological processes in autotrophs, such as photosynthesis, respiration, carbohydrate degradation, and photo respiration, the remarkable increase in the ratio of glucose to glycine may also occur in other phytoplankton species. In addition, we observed alteration of cell size, chlorophyll fluorescence intensity, appearance (color) of culture medium (Fig. 1c, Supplementary Figs. 1 and 2); however, these were insufficient for determining the late stationary phase. This indicated that a metabolic approach provided better resolution of the processes associated with chronological aging of dinoflagellates, as well as in diatoms.

The relative proportions of intracellular free glutamine to glutamate have been used to diagnose N limitation in microalgal cells^35^; however, the ratio fluctuated diurnally, as these amino acids were closely related to N intake and photosynthesis^36^. Unfortunately, we could not assess the validity of the index, as glutamine was not detected in our gas chromatography mass spectrometry (GC-MS) system. While we did not investigate diurnal changes in glucose/glycine, this ratio may change with the circadian rhythm, as the amount of glucose is linked to photosynthesis and respiration. To minimize diurnal changes, we have sampled at a fixed time in this study, that is, 2−4 h after the onset of illumination (9:00–11:00 AM). Nonetheless, future studies are necessary to investigate changes in the criterion with circadian rhythm.

With regard to the practical applicability of using glucose/glycine ratio to predict bloom demise by identifying the late stationary phase of the bloom-forming species, this criterion can only be applied during a bloom year^10^, when only a few plankton species predominate the marine environment and form red tides. In addition, it should be noted that a large variation in this index among the different nutrient statuses may result in lowered confidence in the accuracy of the index; the index in the late stationary phase of the P-dep culture was lower than that in the early stationary phase or decline phase of the N-dep cultures (Fig. 5d–f). However, this apparent shortcoming of the index can be resolved by monitoring the glycine concentrations between the treatments: glycine concentrations in the P-dep culture in the late stationary phase are substantially higher than those of the N-dep culture over the growth curve (Fig. 5b, c). Evidently, because the N and P concentrations in the NP-rep culture are exceptionally higher than those in real-world settings, it is unnecessary to consider the thresholds of the index in the NP-rep culture. Although further investigations are required regarding the practical usefulness of the index, the criterion identified here is a potential candidate for predicting bloom demise and adopting available mitigation measures for minimizing damage and sustainable development of the aquaculture industry.

Finally, the use of the ratio of two metabolite levels for diagnosis indicates that the criterion can be obtained without counting the number of cells, as cell numbers do not need to be factored into the calculations and the amount alone are considered. This implies that bloom demise can be predicted without counting cells, which will save time and labor for phytoplankton observation and thereby eliminate the need for experts in algal biology.

## Methods

### Cell culture and counting of *Karenia mikimotoi*

The unialgal cultures of the dinoflagellate strain of *K. mikimotoi* Km6Y were grown and maintained for several years in our laboratory in modified SWM3 medium based on natural seawater (Supplementary Table 3) at a salinity of 30, under a 12/12 h light/dark cycle of ~600 µmol of photons m^−2^ s^−1^ using white LED illumination at 25 °C. Three different nutrient conditions, NP-rep, N-dep, and P-dep, were prepared. The final concentration of additive nitrogen (NaNO_3_) and phosphate (NaH_2_PO_4_ H_2_O) in the SWM3 medium were 2 and 0.1 mM for NP-rep, 0.2 and 0.1 mM for N-dep (1/10th N of NP-rep), and 2 and 0.0033 mM for P-dep (1/30th P of NP-rep). The experiment was performed using four replicates per treatment. At the start of the culture incubation, the inoculation volumes were 1% and the initial concentration was 1.3 × 10^2^ cells per milliliter.

Cell number were measured for up to 82 days (NP-rep) and 47 days (N-dep and P-dep treatment) at intervals of 4−9 days (day 0, 5, 10, 14, 19, 26, 33, 42, 47, 54, 61, 68, 75, and 82) until the dinoflagellate cells entered the culture demise phase (Fig. 1a), while cell size and chlorophyll fluorescence intensity were investigated for up to 75 days (NP-rep) and 42 days (N-dep and P-dep treatment). For counting *K. mikimotoi* cells, 100 µL was sampled from each culture bottle and fixed using 0.01% glutaraldehyde solution. Immediately after fixation, cells were counted within 10 min using Tali^®^ Image-based Cytometer according to the manufacturer’s instructions (Thermo Fisher Scientific K.K. Tokyo, Japan). Cells with chlorophyll fluorescence were counted using the red (excitation filter, 543/22 nm; long-pass emission filter, 585 nm) channels of the image-based cytometer. The cell size range (in a bright field), red fluorescent threshold, circularity, and sensitivity were set at 16–36 μm, 1500, 8, and 9, respectively^37^. The number of cells was counted in duplicate for each sample, and cell numbers were determined based on their mean values.

### Cell culture and counting of *Chaetoceros tenuissimus*

Separate experiments were performed with the axenic clonal diatom strain, *C. tenuissimus* NIES-3715^38^, to confirm the validity of key criteria in different algal species. Five replicates of the diatom cells were cultured under the NP replete condition. The initial inoculation volume was 0.2% and the cell concentration at the beginning of the experiment was 4.0 × 10^3^ cells per milliliter. The algal strains were cultured in NP-rep SWM3 medium under the same condition as *K. mikimotoi*, and cultures in the late-exponential growth phase were used for inoculations.

For cell counting, a 25 μL aliquot of culture was placed on a disposable counting slide (Thermo Fisher Scientific). After incubation for 10 min in the dark at 20 °C, the cells were counted in the same manner as that for *K. mikimotoi* except that size range was set at 3–20 μm and the red fluorescent threshold at 1200^39^. Cell numbers were counted for up to 28 days at intervals of 5−8 days (day 0, 2, 7, 13, 21, and 28). Glucose and glycine concentrations in each phytoplanktonic cell were not quantified for the metabolomics study of the diatom.

### Growth parameters

Growth rate of microalgae is useful for estimating its physiological state as it reflects the condition of cell division^23^. Several studies have used specific growth rate (SGR) according to the equation: μ_t2_ = (lnx_2_ − lnx_1_)/(t_2_ − t_1_), where x_1_ and x_2_ are the cell numbers at times t_1_ and t_2_, respectively, and μ_t2_ represents μ at t_2_^23,40^. In the present study, the PGR, μ, was estimated using the following equation: μ_t1_ = (lnx_2_ − lnx_1_)/(t_2_ − t_1_). This indicates that PGR aimed to assess potential growth activity of cultured cells at t_1_ until they reached t_2_, unlike SGR, which addresses microalgal activity at t_2_ between days (t_2_−t_1_).

### Sample preparation for metabolomics

Sampling for metabolomics was performed between 9: 00 AM and 11: 00 AM (2–4 h after the onset of illumination) on days 5, 14, 26, 33, 42, 47, 54, 61, 68, and 75 for NP-rep; on days 5, 14, 26, 33, and 42 for N- and P-rep treatments for *K. mikimotoi* (Fig. 1a, b); and on days 2, 7, 13, 21, and 28 for *C. tenuissimus* (Fig. 6a, b).

A sample of each cell culture (10–200 mL, corresponding to approximate 1.1 × 10^5^ – 4.8 × 10^6^ cells and 1.8 × 10^5^ – 6.0 × 10^7^ cells for *K. mikimotoi* and *C. tenuissimus*, respectively) from the culture bottle was retained on a 0.4 μm polycarbonate membrane filter (PC MB 47 mm; GE Healthcare Japan, Tokyo, Japan). The filter retaining the phytoplankton cells was embedded in a glass Petri dish on ice and treated with 750 µL methanol to stop metabolic processes in the cells. After addition of 75 µL ribitol solution (100 mg per liter dissolved in ultrapure water) to the sample, the cells on the filter were resuspended in the methanol-ribitol mixture using a polypropylene cell scraper (AS ONE 2-1994-01; Osaka, Japan). In total, 660 μL of the solution was dispensed into 2 mL micro centrifuge tube. The mixtures were frozen with liquid nitrogen and stored at –85 °C until further analysis.

The phytoplankton cells in solvent mixture (660 µL) were mixed with 240 µL ultrapure water and 240 µL chloroform. Consequently, the metabolites were dissolved in 1080 µL mixture (methanol: ultrapure water: chloroform = 600 µL: 240 µL: 240 µL) and 60 µL ribitol. After vortexing for 10 s, the samples were shaken for 30 min at 37 °C. Then, the mixtures were mixed with 400 µL ultrapure water and centrifuged for 5 min at 16,000 × *g* at 4 °C. The supernatant (800 µL) was mixed with 50 µL d_27_-myristic acid solution (200 mg per liter dissolved in methanol) and transferred to a 1.5-mL Eppendorf tube with a pierced cap. The samples were dried completely in a vacuum centrifuge drier at 30 °C (CVE-3000; Tokyo Rikakikai, Tokyo, Japan). Derivatization was performed in two steps: oximation and silylation. For oximation, 50 µL methoxyamine hydrochloride in dehydrated pyridine (20 mg per milliliter) was added to the samples. The mixture was incubated at 30 °C for 90 min. Thereafter, 25 µL MSTFA was added to the samples and incubated at 37 °C for 30 min for silylation. In all analysis, ribitol was used as an internal standard and d_27_-myristic acid was used to lock the retention time.

The chemicals used in the metabolomics study were as follows: methoxyamine hydrochloride and d_27_-myristic acid from Sigma Aldrich (St. Louis, MO, USA), pyridine from Kanto Kagaku Chemical (Tokyo, Japan), pesticide analytical grade chloroform, HPLC analytical grade methanol, ultrapure water and ribitol from Wako Pure Chemical Industries (Osaka, Japan), and N-methyl-N-(trimethylsilyl)-trifluoroacetamide (MSTFA) from GL Science Inc. (Tokyo, Japan).

### Analytical condition

A 6890 N gas chromatograph system (Agilent Technologies, Tokyo, Japan) equipped with a quadrupole mass spectrometer (Agilent 5975) and a DB-5ms (i.d. 0.25 mm × 30 m, 0.25 µm thickness, 10 m DG; Agilent) was used to perform gas chromatographic separation of the metabolites. Briefly, samples (1 µL) were injected in split mode (10:1, v/v). The temperatures of the injector, transfer line, and ion source were 250, 280, and 250 °C, respectively. Total ion current spectra were recorded in the scan range of *m/z* 50–550. The helium gas flow rate was adjusted to ~1.1 mL/min for d_27_-myristic acid retention time of 16.727 min. The oven temperature program was as follows: 60 °C for 1 min, increased at the rate of 10 °C/min to 325 °C, and held at 325 °C for 10 min.

### Quantification of glucose and glycine in phytoplanktonic cells

Following the diagnostic analysis of metabolites, the concentration of glucose and glycine in dinoflagellate cells was determined. For quantification of glucose and glycine in phytoplanktonic cells, first 60 mg per liter glucose and glycine stock solutions were prepared. Graded concentrations of standard solution in 240 µL ultrapure water were subsequently prepared by diluting the stock solutions with ultrapure water to 0, 3.2, 6.3, 13, and 25 mg per liter. These concentrations corresponded to 0.018, 0.034, 0.069, 0.14 mM for glucose and 0.042, 0.083, 0.17, and 0.33 mM for glycine. The standard solution was mixed with 60 µL ribitol solution, 600 µL methanol, and 240 µL chloroform, followed by extraction and derivatization as described above. Ions with *m/z* 319 for glucose and 174 for glycine were monitored using the GC-MS metabolomics system.

### Data processing and biomarker exploration

During data processing, Mass Hunter (ver.10.1; Agilent Technologies, Tokyo, Japan) was used to assist in ion chromatographic deconvolution. Metabolites were identified and normalized on the basis of their mass spectral patterns and retention times in the Fiehn library (Agilent) as implemented in Mass Hunter. The exclusive masses of each metabolite peak registered in the Fiehn library were normalized using the exclusive mass of the ribitol peak (*m/z* 217) and cell numbers. The peak area of metabolites, which had a signal-to-noise ratio of less than 1, was assigned the value 5 and normalized by the ribitol peak for subsequent statistical analysis. MetaboAnalyst 5.0^41^ was used to investigate differences in the metabolic content among the specified experimental groups.

The methodology for determining the diagnostic metabolic features associated with the late stationary growth phase of *K. mikimotoi* cells is shown in Supplementary Fig. 5. First, candidate metabolites that were robustly detected in phytoplanktonic cells with mean S/N ratio higher than 20 per 1.0 × 10^6^ cells per milliliter were selected for both treatment groups and sampling date. ROC analysis was performed with six selected metabolites individually between the two specified groups: Group I; 5, 14, 26, 33, 54, 61, 68, 75 days for NP-rep and 5, 14, and 42 days for N or P-dep treatment groups; Group II; 42 and 47 days for NP-rep and 26 and 33 days for N or P-dep treatment groups. Group I corresponds to the exponential, early stationary, and decline phases, and Group II to the late stationary phase. This was followed by generalized linear model (glm) based on logistic regression, assuming that the response variable, Y, which was set to physiological state as determined by onset of decline (Group I = 0, Group II = 1), followed binomial distribution^42^. Finally, AUC and its 95% confidence interval (CI), sensitivity, specificity, and accuracy were calculated. Values exceeding 0.7 were considered satisfactory. Computation of ROC analysis was performed using the R software with “pROC” library^43^.

### Statistics and reproducibility

All data shown are presented as the mean and S.D. from four (*K. mikimotoi*) or five (*C. tenuissimus*) biological replicates. Differential metabolites with statistically significant differences between groups were identified using *t*-tests for parametric data and Welch’s *t*-tests for non-parametric data after FDR adjustment at a significance threshold of 0.05 (*q* < 0.05) using MS-DIAL^44^^.^. Differences in cell size and chlorophyll red fluorescence intensity between the NP-rep and the nutrient deficient group on the same sampling date were analyzed using *t*-tests or Welch’s *t*-tests at *p* < 0.05. The criterion for the diagnosis of stationary phase was assessed between Group I and Group II using the *F*-test (checking homogeneity of variances), followed by two-sample *t*-test.

### Reporting summary

Further information on research design is available in the Nature Portfolio Reporting Summary linked to this article.

## Supplementary information

Supplementary Information

Description of Additional Supplementary Files

Supplementary Data 1

Reporting Summary

## Data Availability

Source data underlying Figs. 1a, b, 2a–c, 3a, b, 4, 5, 6, Table 1, Supplementary Tables 1, 2, and Supplementary Figs. 1, 2, 3 and 4 are provided as Supplementary Data 1. All other data generated during this study are available from the corresponding author on reasonable request.
