# Supplementary material for: Chronological age-related metabolome responses in the dinoflagellate Karenia mikimotoi, can predict future bloom demise
Source: Commun Biol. 2023 Mar 15;6:273. doi: 10.1038/s42003-023-04646-z (PMC10017670; doi:10.1038/s42003-023-04646-z)
Supplement: Supplementary file 1 — Supplementary Information [file 42003_2023_4646_MOESM1_ESM.docx]

Supplementary Information for:

**Chronological age-related metabolome responses in the dinoflagellate *Karenia mikimotoi*, can predict future bloom demise**

Takeshi Hano^*^, Yuji Tomaru

Environment Conservation Division, Fisheries Technology Institute, National Research and Development Agency, Japan Fisheries Research and Education Agency,

2-17-5 Maruishi, Hatsukaichi, Hiroshima 739-0452, Japan

*Corresponding author

National Research Institute of Fisheries and Environment of Inland Sea, Fisheries Research and Education Agency, 2-17-5, Maruishi, Hatsukaichi, Hiroshima Japan, 739-0452

Tel: +81-829-55-0666; Fax: +81-829-54-1216; E-mail address: hano_takeshi74@fra.go.jp

In this document, 3 tables and 5 figures.

**Supplementary Table 1. Sensitivity, specificity, accuracy, and AUC (95% CI) of metabolites.**


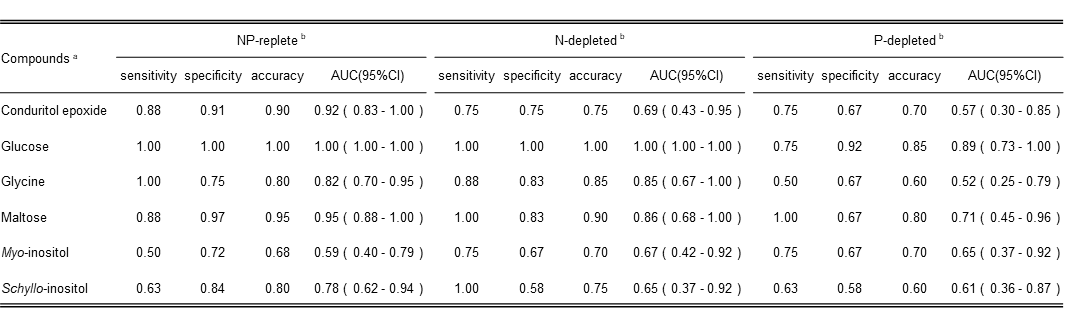


^a^: Of the 46 metabolites tested, only those that were mean signal-to-noise ratio (S/N ratio) being higher than 20 per 1 × 10^6^ cells per milliliter for any treatment group and sampling date were included in the table.

^b^: AUC, area under the curve; CI, credible interval.

**Supplementary Table 2. Sensitivity, specificity, accuracy, and AUC (95% CI) of the ratio of glucose to the other five metabolites.**

**
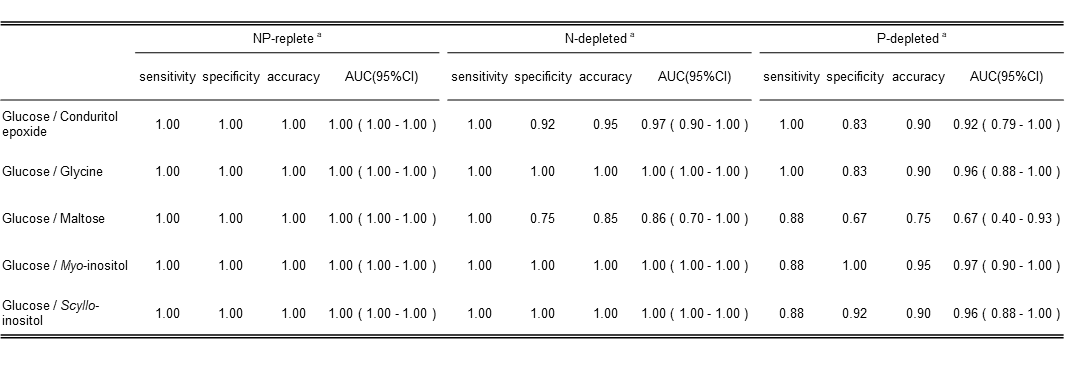
**

^a^: AUC, area under the curve. CI, credible interval.

**Supplementary Table 3. List of chemicals used for modified SWM-3**

|  | Stock solution | Final concentrations in 1000 mL | | |
| --- | --- | --- | --- | --- |
|  |  | NP replete | N depleted | P depleted |
| NaNO_3_  NaH_2_PO_4_ 2H_2_O  Na_2_SiO_3_ 9H_2_O  Na_2-_EDTA  Fe-EDTA  Na_2_SeO_3_  Tris  P-1 Metal solution ^a^  S3 Vitamin Sol. ^b^  Sea water  pH 7.7−7.8 | 21.25 g/250 mL  1.95 g/250 mL  7.105 g/250 mL  1.40 g/250 mL  0.105 g/250 mL  0.346 mg/L | 2.0 mM  0.1 mM  0.2 mM  30 µM  2 µM  2 nM  500 mg  10 mL  2 mL  Up to 1000 mL | 0.2 mM  0.1 mM  0.2 mM  30 µM  2 µM  2 nM  500 mg  10 mL  2 mL  Up to 1000 mL | 2.0 mM  0.003 mM  0.2 mM  30 µM  2 µM  2 nM  500 mg  10 mL  2 mL  Up to 1000 mL |

^a^

| P-1 Metal solution | in 1000 mL |
| --- | --- |
| H_3_BO_3_  MnCl_2_ 4H_2_O  ZnCl_2_  CoCl_2_ 6H_2_O | 6.183 g  692.5 mg  54.5 mg  2.38 mg |

^b^

| S3 Vitamin solution | in 1000 mL |
| --- | --- |
| Thiamine HCl  Nicotinic acid  Ca pantothenate  p-aminobenzoic acid  Biotin  Inositol  Folic acid  Thymine  B12 | 0.25 g  0.05 g  0.05 g  5 mg  0.5 mg  2.5 g  1 mg  1.5 g  1 mg |


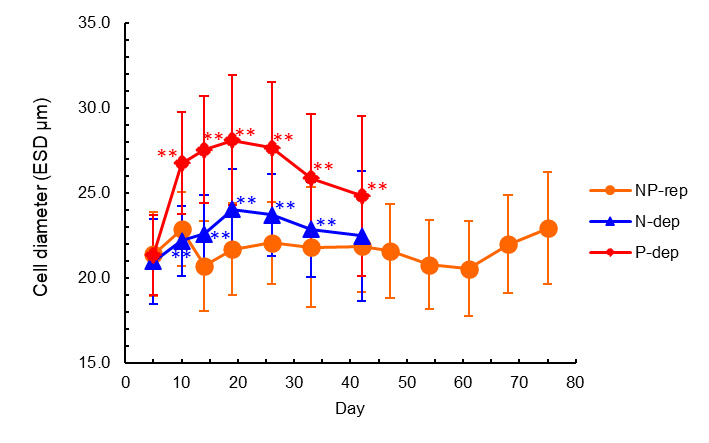
**a**

**b**


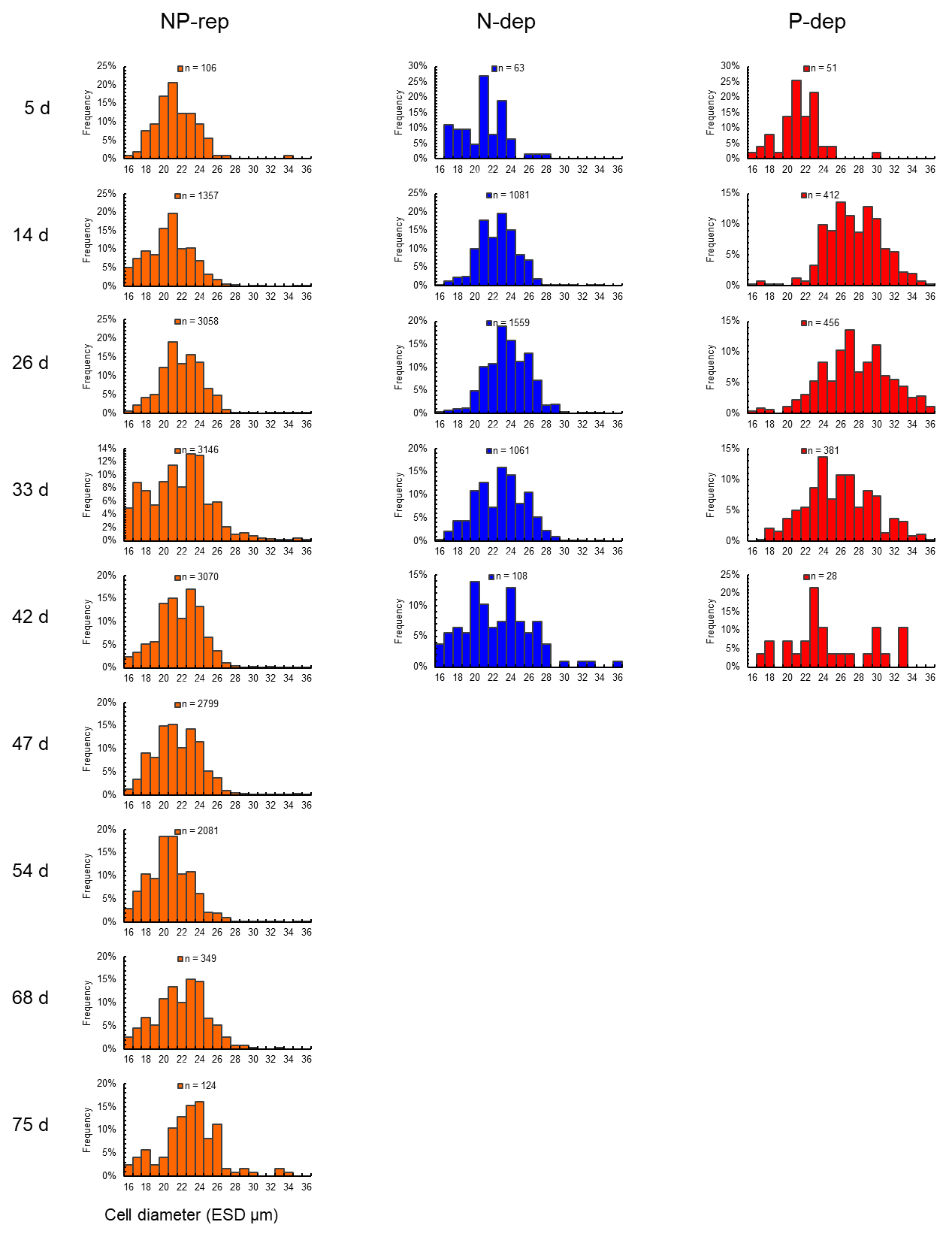


**Supplementary Fig. 1** **Diameter (µm/cell) of *K. mikimotoi* cells. a)** Mean fold changes and **b)** histograms of cell size during the experimental period. The dinoflagellate cells were cultured for 47 or 82 days in the SWM-3 medium with different concentrations of nitrogen (NaNO_3_) and phosphate (NaH_2_PO_4_) and initial N and P concentrations were 2 and 0.1 mM for NP-replete (NP-rep), 0.2 and 0.1 mM for N-depleted (N-dep), and 2 and 0.0033 mM for P-depleted (P-dep) treatment groups. In (**a**), data are expressed as the mean ± standard deviation (n = 4). **, *p < 0.01* versus the NP-replete on the same sampling date (two sample *t-*tests for parametric data and Welch’s t-tests for non-parametric data). Rep., replete; dep., depleted.

**
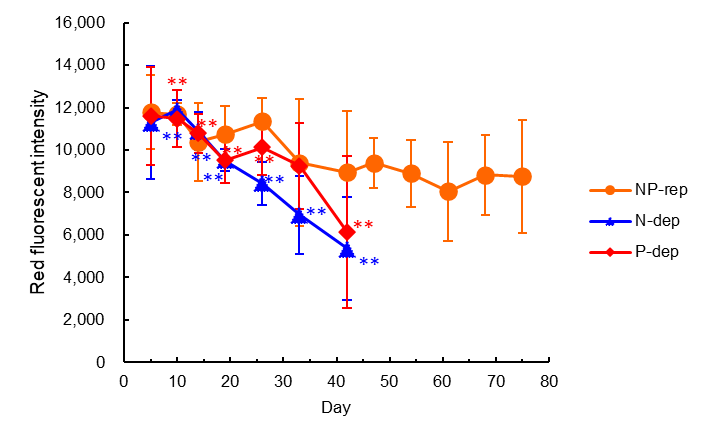
a**

**b**


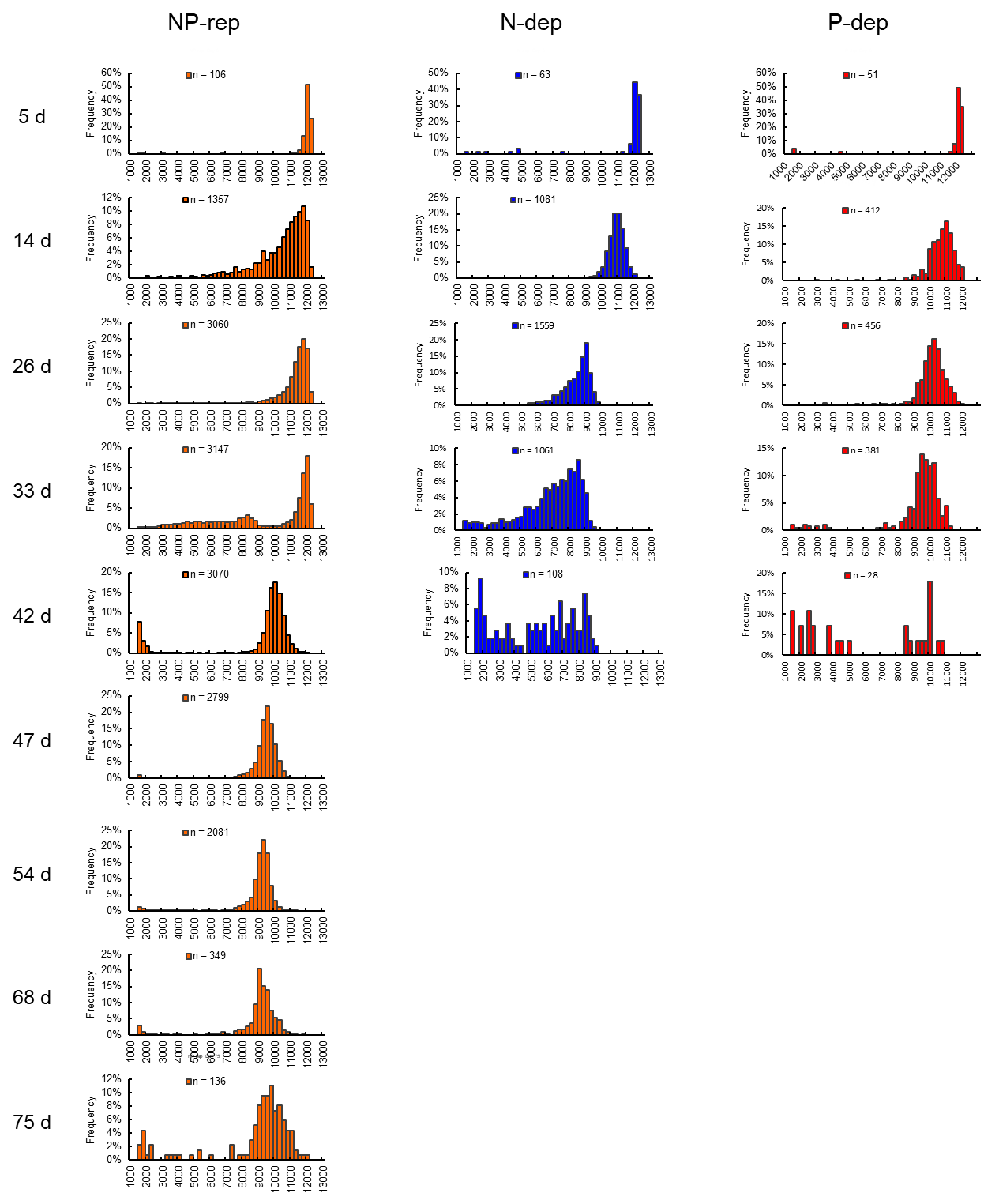


**Supplementary Fig. 2** **Unicellular** **chlorophyll red fluorescence intensity of *K. mikimotoi* cells.** **a)** Mean fold changes and **b)** histograms of red fluorescence intensity during the experimental period. The dinoflagellate cells were cultured for 47 or 82 days in the SWM-3 medium with different concentrations of nitrogen (NaNO_3_) and phosphate (NaH_2_PO_4_) and initial N and P concentrations were 2 and 0.1 mM for NP-replete (NP-rep), 0.2 and 0.1 mM for N-depleted (N-dep), and 2 and 0.0033 mM for P-depleted (P-dep) treatment groups. In (**a**), data are expressed as the mean ± standard deviation (n = 4). **, *p < 0.01* versus the NP-replete on the same sampling date (two sample *t-*tests for parametric data and Welch’s t-tests for non-parametric data). Rep., replete; dep., depleted.


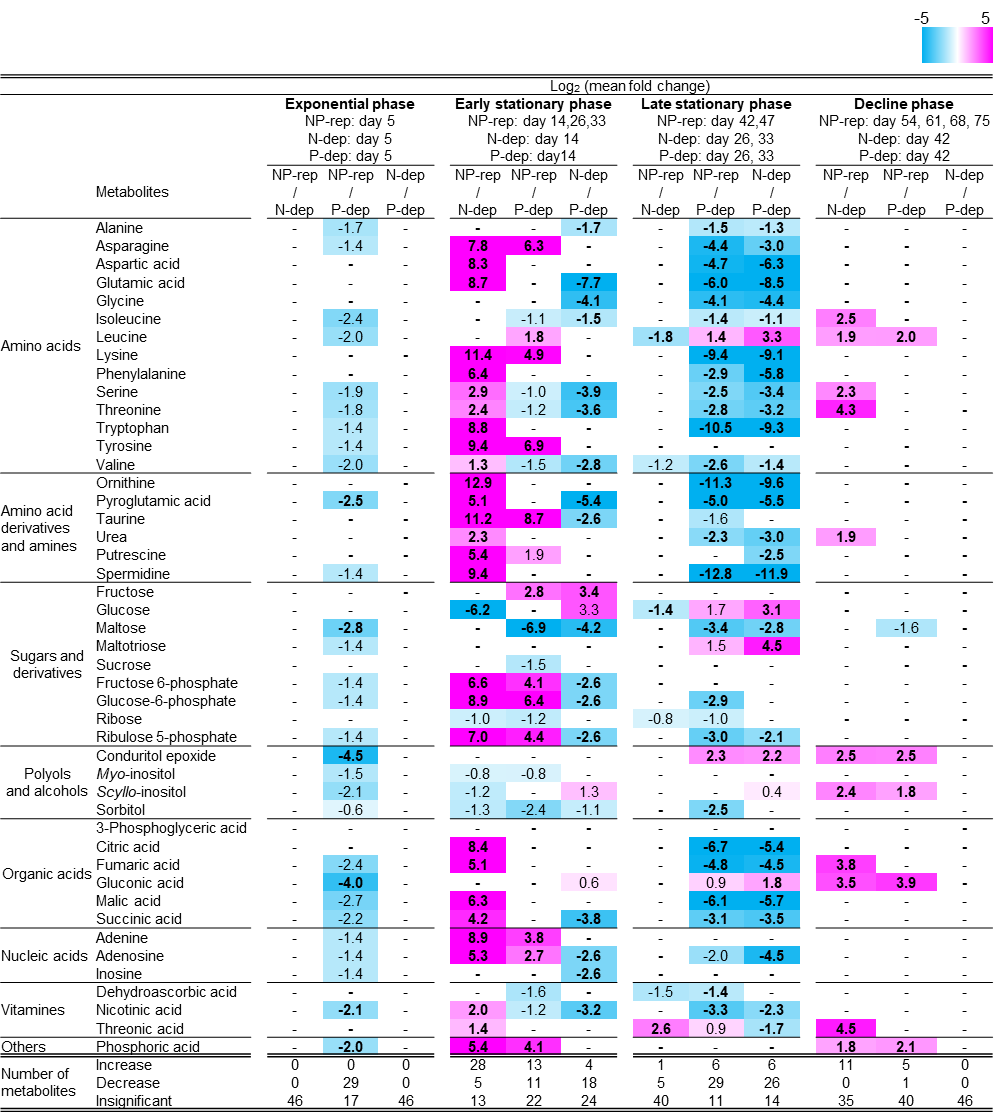


**Supplementary Fig. 3 Heatmap of growth phase-, nutrient- related metabolic changes in the *K. mikimotoi*.** Log_2_-transformed mean fold changes in metabolites among various nutrient media at the specified growth phase. The dinoflagellate cells were cultured for 47 or 82 days in the SWM-3 medium with different concentrations of nitrogen (NaNO_3_) and phosphate (NaH_2_PO_4_) and initial N and P concentrations were 2 and 0.1 mM for NP-replete (NP-rep), 0.2 and 0.1 mM for N-depleted (N-dep), and 2 and 0.0033 mM for P-depleted (P-dep) treatment groups. Plain and bold numbers indicate significant fold changes based on t-tests for parametric data and Welch’s t-tests for non-parametric data, respectively, after FDR adjustment at a significance threshold of 0.05 (*q* < 0.05). Fold changes are color-coded; pink and sky blue indicate significant increase and decrease and “-“denote insignificant changes. Rep., replete; dep., depleted.


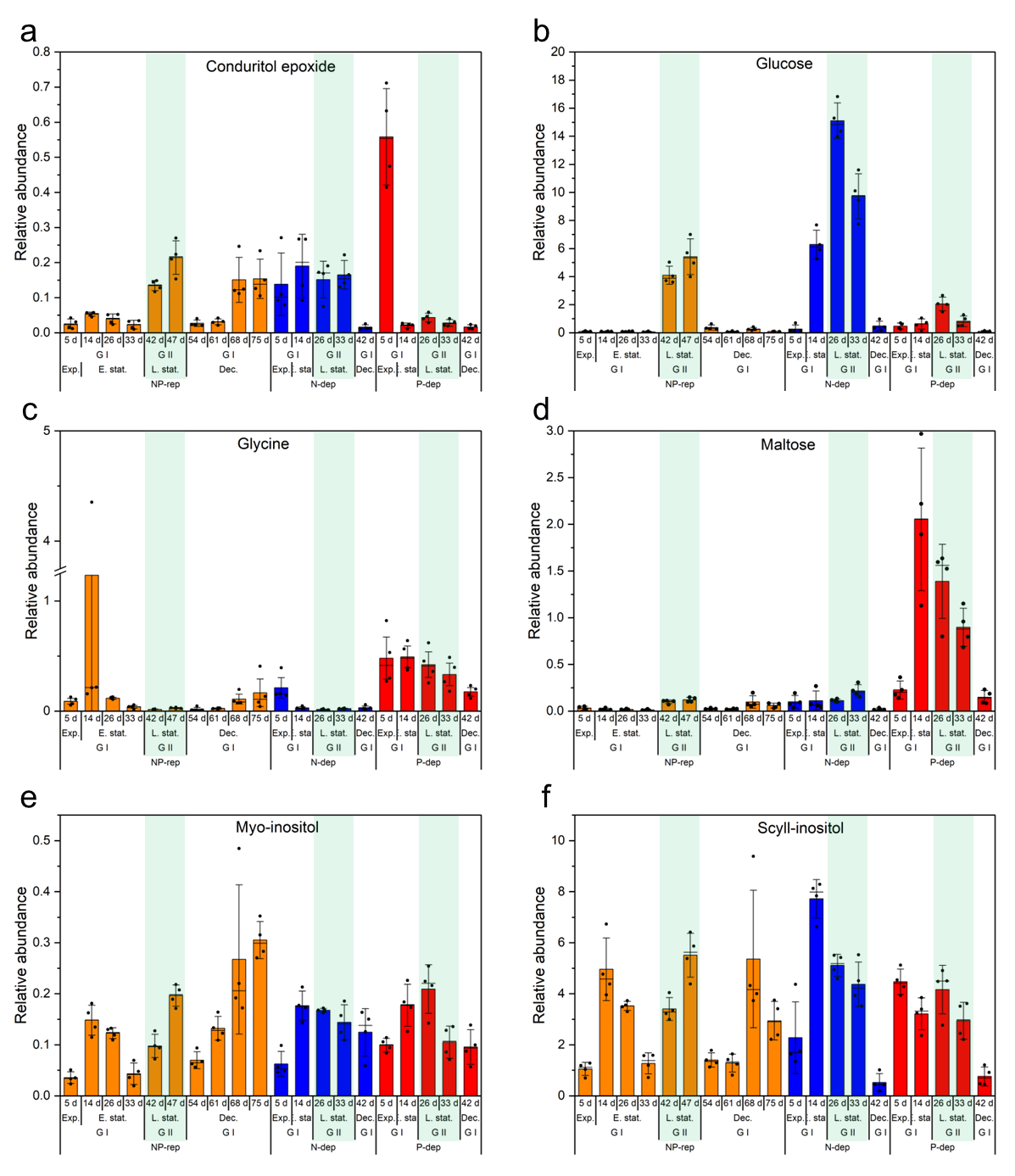


**Supplementary Fig. 4 Chronological alterations of metabolites and criterion in the *K. mikimotoi*.** Of the 46 metabolites tested, each graph represents relative abundance (abundance of metabolite normalized by cell number (1.0 × 10^3^ cells) and internal standard) of six metabolites (**a**-**f**) whose mean signal-to-noise ratio (S/N ratio) being higher than 20 per 1.0 × 10^6^ cells per milliliter for any treatment group and sampling date. The dinoflagellate cells were cultured for 47 or 82 days in the SWM-3 medium with different concentrations of nitrogen (NaNO_3_) and phosphate (NaH_2_PO_4_) and initial N and P concentrations were 2 and 0.1 mM for NP-replete (NP-rep), 0.2 and 0.1 mM for N-depleted (N-dep), and 2 and 0.0033 mM for P-depleted (P-dep) treatment groups. Data indicate mean ± standard deviation (SD; n = 4). Exp., Exponential; E. stat, Early stationary; L. stat, Late stationary; Dec, Decline; G I, Group I; G II, Group II; Rep, replete; dep, depleted. Light green shades in each graph show late stationary phase (Group II).


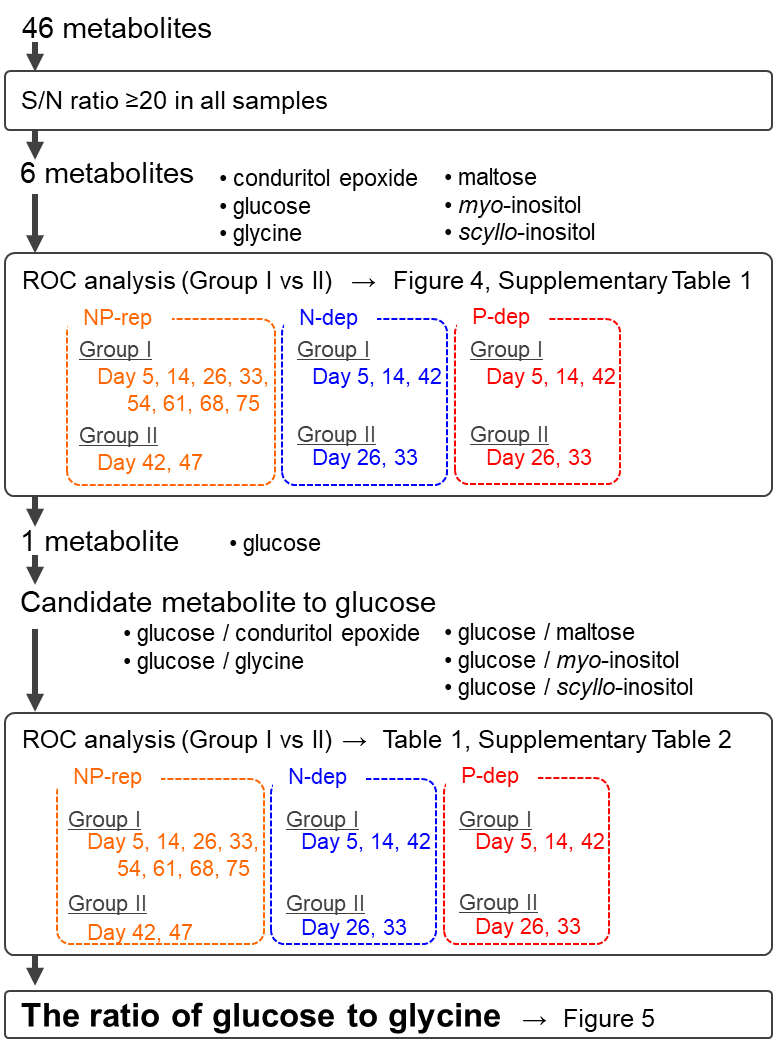


**Supplementary Fig. 5** **Experimental flow showing the criterion for metabolite determination.** ROC., Receiver operating characteristic; Rep., replete; dep., depleted.
