# Supplementary material for: Chronological age-related metabolome responses in the dinoflagellate Karenia mikimotoi, can predict future bloom demise
Source: Commun Biol. 2023 Mar 15;6:273. doi: 10.1038/s42003-023-04646-z (PMC10017670; doi:10.1038/s42003-023-04646-z)
Supplement: Supplementary file 2 — Description of Additional Supplementary Files [file 42003_2023_4646_MOESM2_ESM.pdf]

Description of Additional Supplementary Files

File name: Supplementary Data 1

Description: The source data behind the Figs and Tables in the paper.
