# Supplementary material for: Chronological age-related metabolome responses in the dinoflagellate Karenia mikimotoi, can predict future bloom demise
Source: Commun Biol. 2023 Mar 15;6:273. doi: 10.1038/s42003-023-04646-z (PMC10017670; doi:10.1038/s42003-023-04646-z)
Supplement: Supplementary file 4 — Reporting Summary [file 42003_2023_4646_MOESM4_ESM.pdf]

nature portfolio

Corresponding author(s): Takeshi Hano

Last updated by author(s): Feb 17, 2023

# Reporting Summary

Nature Portfolio wishes to improve the reproducibility of the work that we publish. This form provides structure for consistency and transparency in reporting. For further information on Nature Portfolio policies, see our Editorial Policies and the Editorial Policy Checklist.

## Statistics

For all statistical analyses, confirm that the following items are present in the figure legend, table legend, main text, or Methods section.

| n/a | Confirmed | |
|---|---|---|
| ☐ | ☒ | The exact sample size (*n*) for each experimental group/condition, given as a discrete number and unit of measurement |
| ☐ | ☒ | A statement on whether measurements were taken from distinct samples or whether the same sample was measured repeatedly |
| ☐ | ☒ | The statistical test(s) used AND whether they are one- or two-sided<br>*Only common tests should be described solely by name; describe more complex techniques in the Methods section.* |
| ☒ | ☐ | A description of all covariates tested |
| ☐ | ☒ | A description of any assumptions or corrections, such as tests of normality and adjustment for multiple comparisons |
| ☐ | ☒ | A full description of the statistical parameters including central tendency (e.g. means) or other basic estimates (e.g. regression coefficient) AND variation (e.g. standard deviation) or associated estimates of uncertainty (e.g. confidence intervals) |
| ☐ | ☒ | For null hypothesis testing, the test statistic (e.g. $F$, $t$, $r$) with confidence intervals, effect sizes, degrees of freedom and $P$ value noted<br>*Give P values as exact values whenever suitable.* |
| ☒ | ☐ | For Bayesian analysis, information on the choice of priors and Markov chain Monte Carlo settings |
| ☐ | ☒ | For hierarchical and complex designs, identification of the appropriate level for tests and full reporting of outcomes |
| ☒ | ☐ | Estimates of effect sizes (e.g. Cohen's *d*, Pearson's *r*), indicating how they were calculated |

*Our web collection on statistics for biologists contains articles on many of the points above.*

## Software and code

Policy information about availability of computer code

| | |
|---|---|
| Data collection | No software was used for data collection. |
| Data analysis | For data analysis, we used R software for ROC analysis with "pROC" library. |

For manuscripts utilizing custom algorithms or software that are central to the research but not yet described in published literature, software must be made available to editors and reviewers. We strongly encourage code deposition in a community repository (e.g. GitHub). See the Nature Portfolio guidelines for submitting code & software for further information.

## Data

Policy information about availability of data

All manuscripts must include a data availability statement. This statement should provide the following information, where applicable:

- Accession codes, unique identifiers, or web links for publicly available datasets
- A description of any restrictions on data availability
- For clinical datasets or third party data, please ensure that the statement adheres to our policy

All materials are available from the corresponding author upon reasonable request.

## Human research participants

Policy information about studies involving human research participants and Sex and Gender in Research.

| | |
|---|---|
| Reporting on sex and gender | The present study does not include human research. |
| Population characteristics | The present study does not include human research. |
| Recruitment | The present study does not include human research. |
| Ethics oversight | The present study does not include human research. |

Note that full information on the approval of the study protocol must also be provided in the manuscript.

# Field-specific reporting

Please select the one below that is the best fit for your research. If you are not sure, read the appropriate sections before making your selection.

☐ Life sciences ☐ Behavioural & social sciences ☒ Ecological, evolutionary & environmental sciences

For a reference copy of the document with all sections, see nature.com/documents/nr-reporting-summary-flat.pdf

# Ecological, evolutionary & environmental sciences study design

All studies must disclose on these points even when the disclosure is negative.

| | |
|---|---|
| Study description | This study provide insights regarding chronological aging of harmful dinoflagellate K. mikimotoi under nitrogen or phosphorus deficient condition and identify a key metabolite signature for the late stationary phase, which will allow us to predict future bloom demise. The experiment include two treatment factors including nutrient (NP-replete, N-depleted and P-depleted) and chronological change. We performed the test with four replicates. The result of key metabolite signature identified from K. mikimotoi was also validated by cultured diatom Cheatoceros tennuissimus (five replicates). |
| Research sample | We used K. mikimotoi KmURN16Y strain, which was sampled at Uranouchi Inlet,(N 33.4285, E 133.4001) on Jun 24, 2009. Sequences including the regions of large subunit (LSU), 5.8S, and the internal transcribed spacer (ITS) is LC055223. Details are available at https://doi.org/10.1016/j.hal.2016.04.007<br>For, C. tenuissimus NIES-3715 strain, we purchased from National Institute of Environmental studies, Japan (https://www.nies.go.jp/index-e.html) |
| Sampling strategy | The experiment was performed with four replicates for K. mikimotoi and with five replicates for C. tennuisimus. Actually, we think four is the minimum number of replicates for statistical analysis. In addition, the data showed that there are quite small variations among the replicates, indicating that the experiment was robustly conducted. We therefore believe that four is sufficient in our experiment. |
| Data collection | Both authors (Hano T and Tomaru Y) and our assistants recorded available data in a notebook and electronic data (i.e., mass spectrometry data) in computers. |
| Timing and spatial scale | Data on K. mikimotoi cell were collected from Jan. 17. 2020 from Apr.9.2020.<br>Data on C.tennuissimus were collected from Nov. 8.2016 to Dec.6. 2016.<br>Both experiments were conducted separately, however, we believe it does not affect our conclusions. |
| Data exclusions | No data were excluded from the analyses. |
| Reproducibility | We performed experiments with four and five replicates for K. mikimotoi and C.tennuissimus, respectively. We did not conduct additional experiment to confirm reproducibility. However, considering that cell culture sampling was performed with several times for chronological observations and there are quite small variations among the replicates, we strongly believe that our findings are highly reproducible. |
| Randomization | We randomly allocated phytoplankton into each glass flasks when cell culture was initiated. |
| Blinding | In our experiment with phytoplankton, blinding is impossible because we observed chronological changes of phytoplanton cells cultured in labeled flasks. |

Did the study involve field work? ☐ Yes ☒ No

# Reporting for specific materials, systems and methods

We require information from authors about some types of materials, experimental systems and methods used in many studies. Here, indicate whether each material, system or method listed is relevant to your study. If you are not sure if a list item applies to your research, read the appropriate section before selecting a response.

## Materials & experimental systems

| n/a | Involved in the study |
|---|---|
| ☒ | ☐ Antibodies |
| ☐ | ☒ Eukaryotic cell lines |
| ☒ | ☐ Palaeontology and archaeology |
| ☒ | ☐ Animals and other organisms |
| ☒ | ☐ Clinical data |
| ☒ | ☐ Dual use research of concern |

## Methods

| n/a | Involved in the study |
|---|---|
| ☒ | ☐ ChIP-seq |
| ☒ | ☐ Flow cytometry |
| ☒ | ☐ MRI-based neuroimaging |

# Eukaryotic cell lines

Policy information about cell lines and Sex and Gender in Research

| | |
|---|---|
| Cell line source(s) | We used K. mikimotoi KmURN16Y strain, which was sampled at Uranouchi Inlet,(N 33.4285, E 133.4001) on Jun 24, 2009. Sequences including the regions of large subunit (LSU), 5.8S, and the internal transcribed spacer (ITS) is LC055223. Details are available at https://doi.org/10.1016/j.hal.2016.04.007<br>For, C. tenuissimus NIES-3715 strain, we purchased from National Institute of Environmental studies, Japan (https://www.nies.go.jp/index-e.html) |
| Authentication | K.mikimotoi cell lines are available from the author upon reasonable request. |
| Mycoplasma contamination | We declare that cell lines were not tested for mycoplasma contamination. |
| Commonly misidentified lines<br>(See ICLAC register) | We do not use misidentified cell lines. |
